# Supplementary material for: Iterative improvement in the automatic modular design of robot swarms
Source: PeerJ Comput Sci. 2020 Dec 7;6:e322. doi: 10.7717/peerj-cs.322 (PMC7924708; doi:10.7717/peerj-cs.322)
Supplement: Supplemental Information 3 [file peerj-cs-06-322-s003.zip › argos3/doc/api/standalone/a00411_source.html]

ARGoS: core/wrappers/lua/lua\_utility.h Source File


- Main Page
- Related Pages
- Namespaces
- Classes
- Files

- File List
- File Members

# core/wrappers/lua/lua\_utility.h

Go to the documentation of this file.

```
00001 #ifndef LUA_UTILITY_H
00002 #define LUA_UTILITY_H
00003 
00010 namespace argos {
00011    class CRadians;
00012    class CVector2;
00013    class CVector3;
00014    class CQuaternion;
00015    class CColor;
00016 }
00017 
00018 #include <argos3/core/utility/logging/argos_log.h>
00019 #include <argos3/core/utility/math/rng.h>
00020 
00021 extern "C" {
00022 #include <lua.h>
00023 #include <lualib.h>
00024 #include <lauxlib.h>
00025 }
00026 
00027 #include <string>
00028 
00029 namespace argos {
00030 
00031    class CLuaUtility {
00032 
00033    public:
00034 
00035       enum EARGoSTypes {
00036          TYPE_NORMAL = 0,
00037          TYPE_VECTOR2,
00038          TYPE_VECTOR3,
00039          TYPE_QUATERNION,
00040          TYPE_COLOR
00041       };
00042       
00043    public:
00044 
00051       static bool LoadScript(lua_State* pt_state,
00052                              const std::string& str_filename);
00053       
00060       static bool CallLuaFunction(lua_State* pt_state,
00061                                   const std::string& str_function);
00062 
00071       static void PrintGlobals(CARGoSLog& c_log,
00072                                lua_State* pt_state);
00073       
00082       static void PrintStack(CARGoSLog& c_log,
00083                              lua_State* pt_state);
00084 
00094       static void RegisterLoggerWrapper(lua_State* pt_state);
00095 
00105       static void RegisterRNG(lua_State* pt_state,
00106                               CRandom::CRNG* pc_rng);
00107 
00117       static void OpenRobotStateTable(lua_State* pt_state,
00118                                       const std::string& str_key);
00119 
00126       static void CloseRobotStateTable(lua_State* pt_state);
00127 
00136       static void StartTable(lua_State* pt_state,
00137                              const std::string& str_key);
00138 
00147       static void StartTable(lua_State* pt_state,
00148                              int n_key);
00149 
00157       static void EndTable(lua_State* pt_state);
00158 
00167       static void AddToTable(lua_State* pt_state,
00168                              const std::string& str_key,
00169                              void* pt_data);
00170 
00179       static void AddToTable(lua_State* pt_state,
00180                              const std::string& str_key,
00181                              lua_CFunction pt_data);
00182 
00191       static void AddToTable(lua_State* pt_state,
00192                              const std::string& str_key,
00193                              Real f_data);
00202       static void AddToTable(lua_State* pt_state,
00203                              const std::string& str_key,
00204                              const std::string& str_data);
00205 
00214       static void AddToTable(lua_State* pt_state,
00215                              int n_key,
00216                              Real f_data);
00217 
00226       static void AddToTable(lua_State* pt_state,
00227                              const std::string& str_key,
00228                              const CRadians& c_data);
00229 
00238       static void AddToTable(lua_State* pt_state,
00239                              int n_key,
00240                              const CRadians& c_data);
00241 
00252       static void AddToTable(lua_State* pt_state,
00253                              const std::string& str_key,
00254                              const CVector2& c_data);
00255       
00266       static void AddToTable(lua_State* pt_state,
00267                              int n_key,
00268                              const CVector2& c_data);
00269       
00280       static void AddToTable(lua_State* pt_state,
00281                              const std::string& str_key,
00282                              const CVector3& c_data);
00283       
00294       static void AddToTable(lua_State* pt_state,
00295                              int n_key,
00296                              const CVector3& c_data);
00297       
00308       static void AddToTable(lua_State* pt_state,
00309                              const std::string& str_key,
00310                              const CQuaternion& c_data);
00311       
00322       static void AddToTable(lua_State* pt_state,
00323                              int n_key,
00324                              const CQuaternion& c_data);
00325       
00336       static void AddToTable(lua_State* pt_state,
00337                              const std::string& str_key,
00338                              const CColor& c_data);
00339       
00350       static void AddToTable(lua_State* pt_state,
00351                              int n_key,
00352                              const CColor& c_data);
00353 
00364       template<class T>
00365       static T* GetDeviceInstance(lua_State* pt_state,
00366                                   const std::string& str_key) {
00367          lua_getglobal(pt_state, "robot");
00368          lua_getfield(pt_state, -1, str_key.c_str());
00369          lua_getfield(pt_state, -1, "_instance");
00370          T* ptRetVal = reinterpret_cast<T*>(lua_touserdata(pt_state, -1));
00371          lua_pop(pt_state, 3);
00372          return ptRetVal;
00373       }
00374       
00375    private:
00376       
00377       static int LOGWrapper(lua_State* pt_state);
00378       
00379       static int LOGERRWrapper(lua_State* pt_state);
00380 
00381       static int LoggerWrapper(CARGoSLog& c_log,
00382                                lua_State* pt_state);
00383 
00384    };
00385 
00386 }
00387 
00388 #endif
```

---

Generated on 10 Jul 2018 for ARGoS by 
 1.6.1
